# Social Network Types and Depressive Symptoms among Older Korean Men and Women

**DOI:** 10.3390/ijerph182111175

**Published:** 2021-10-24

**Authors:** Kyung-Won Choi, Gyeong-Suk Jeon

**Affiliations:** Department of Nursing, Korea National University of Transportation, Chungju 27909, Korea; kwchoi@ut.ac.kr

**Keywords:** social network types, depressive symptoms, older adults

## Abstract

This study explores the social network types of older Korean men and women, and the relationship of those networks to depressive symptoms. A population of 4608 older adults who participated in the Living Profiles of Older People Survey (LPOPS) were included in the study. Seven criterion variables—marital status, living arrangements, frequency of contact with children, close friends, and close relatives, participation in social activities, and total network size—were included in a K-means cluster analysis. Multivariable logistic regression analysis of the impact of social network type on depressive symptoms was conducted. We identified two “diverse type” social networks (diverse-married and diverse-unmarried) in women, and one diverse type and one “social-activity-focused type” network in men. Family focused type and two “restricted type” social networks (restricted-couple-focused, and restricted-unmarried) were identified in both men and women. The restricted-unmarried and restricted-couple-focused networks were associated with more depressive symptoms in both men and women. However, the family focused type was associated with more depressive symptoms only in women. The results indicated that social network types, and their impact on depressive symptoms, differ by gender. There is a need for further research on gender differences in the social network types of older adults across diverse cultures.

## 1. Introduction

A social network can be conceived of as a web of social relationships [1] allowing for the transmission of information and providing social support [2]. Social networks are crucial for successful ageing. A considerable body of research has found that maintenance of social relations in old age is important to prevent depression, functional decline, and mortality, and to maintain cognitive function [3,4,5,6].

Individuals are embedded within social networks [7]; this has implications for health, as described above [8]. The network types and prevalent network types have been different across countries and cultures. Social network typology studies in Europe, North America, and Israel have identified four robust network types based on size of social relations and frequency of contact—diverse, family focused, friend-focused, and restricted [7,9,10,11]. The diverse and friend-focused networks constituted over half of older adults in Israeli (71.3%) and American (55.9%) populations, and the family network was the least prevalent. These types are differentially associated with morbidity, subjective health status, depression, subjective psychosocial well-being. Those with a wider social relations such as diverse and friend focused network types report better health and psychological wellbeing than those in more restricted network types [7,9,10,11]. Cheng and colleagues focused on the nature of kinship among older adults in Hong Kong, and identified unique “distant family” network type with four common network types found in America, Israel, and Europe [12]. Diverse (26%) and friend-focused networks (25.6%) were also prevalent in Hong Kong as well as in western societies, and life satisfaction was highest among those in diverse and family focused networks. In Japan, a unique type of “married and distal” network type was identified in addition to four similar above mentioned network types [13]. The diverse network type was uncommon (17%), while the family focused network was the most prevalent in this population (29%). Network types in older adults in Japan did not differ in depressive symptoms, while those in restricted networks showed the highest levels of depressive symptoms and negative effect in China [12].

Park et al. [14] found two diverse types (diverse-family, diverse-friend), friend-focused type and two restricted types (distant, restricted) in Korean older population. A more recent Korean study [15] identified socially integrated types (diverse-couple, diverse-family, congregant) and socially isolated types (restricted-married and widowed). Family focused types were the least prevalent (15% [14], 14.5% [15]) and restricted/isolated types were the most prevalent (47% [14], 24.9% [15]) in both studies with Korean older population. Meanwhile, in older population of Japan and China, prevalence of family focused type was higher than that of restricted type [12,13].

The gendered nature of social roles influences social network characteristics such as relationship with children, friends, and relatives, social participation, and so on. Women tend to have more sources of support, such as children, friends, and neighbors, and to benefit more from them. Women generally take the lead in child-rearing, which is associated with participation in new types of social networks centered around childcare facilities, playgroups, and other informal associations. Thus, women tend to maintain social ties and friendships in their later years. Older women living alone are not at risk for increased mental health or for isolation from social network and social engagement when there are no financial difficulties [16,17]. In contrast, men are more likely to have social networks based primarily on workplace relationships and tend to rely on their spouses for emotional support [1,18]. Men experience network reduction in retirement and suffer more distress with loss of their spouse than women, particularly due to burden of domestic chores and lack of emotional support and social network, such as church attendance and participation of social activities [19]. Jeon and colleagues [20] reported that living alone was associated with a three-to-six-fold increase in the risk of depressive symptoms among Korean older men but not among women. Gendered pattern of mental health among those living alone was more strongly seen in Korean older population than in western population. In western older population, after adjusting for lack of social support, household income and no health limitations, the relationship between living alone and psychological health did not differ between older men and women [21,22,23]. Older adults living alone, mainly women, form an increasing group in many countries as well as Korea. In 2020, approximately, 19.6% of older population in South Korea were single households, and this percentage has increased from 16.0% in 2000. Among those, over 70% were older women [24]. Based on older women’s overwhelming percentage in single households, social network typology needs to be classified by gender, because there are differences in the number and type of networks for support and the impact of living alone between men and women.

Based on evidence from the Western and Asian countries, we could infer that social network types and the relationship between mental health and social networks differs according to cultural values, social circumstances, and gender. However, to our knowledge, little research has been conducted on social network types and its impact on mental health by gender among older adults in South Korea. Therefore, the current study investigated this issue in a large, nationally representative sample of older Koreans. We hypothesized that social network types would differ by gender, as would their association with mental health. The goal was to improve our understanding of the link between social network types and mental health in old age.

## 2. Materials and Methods

### 2.1. Participants

Data from the Living Profiles of Older People Survey (LPOPS), conducted by the Korean Ministry of Health and Welfare in 2017, were analyzed in this study. The LPOPS used a two-stage stratified cluster sampling method; older residents were selected from households in 25 metropolitans and provincial (urban and rural) regions. After receiving approval, trained research staff visited participants in their homes and obtained informed consent. A total of 10,299 participants completed in-person interviews. Older adults with cognitive impairment (1394 men and 3316 women), physical dependence (733 men and 2169 women), or interviews completed by proxy (*n* = 216) were excluded. This was due to concerns that mobility and cognitive function may influence the ability to maintain social networks [25,26], and that the responses of proxies may not fully reflect the views of participants. Ultimately, a weighted population of 4608 participants aged 65 years or older (2384 men and 2224 women) were included in the analysis. The mean age of women was 71.63 years (±5.05), and that of men was 72.93 years (±5.50). Cognitive function was measured using the Mini-Mental State Examination for Dementia Screening (MMSE-DS) instrument [27]. Physical limitations, defined as being dependent on others for one or more activities of daily living, were measured with the Korean version of the Instrumental Activities of Daily Living scale (K-IADL) [28]. The study was approved by the Institutional Review Board of Mokpo University (MNUIRB-210312-SB-005-01).

### 2.2. Measures

#### 2.2.1. Social Networks

Following previous studies on social network topology [10,13,14,15], social network types were distinguished based on seven criterion variables: marital status, living arrangements, frequency of contact with children, frequency of contact with close friends, frequency of contact with close relatives, frequency of participation in social activities, and total network size. Marital status was coded dichotomously (0 = not married, 1 = married or living with partner), as was living arrangements (0 = living alone, 1 = living with others). Respondents who lived with their spouses or partners were considered married, while those who were divorced, separated, widowed, or never married were categorized as not married. Contact frequency was determined based on face-to-face and telephone contact in the past year (0 = never, 6 = almost every day). The average score for these two types represented the frequency of contact. Frequency of contact with children was determined based on contact frequency with both coresiding and non-coresiding children, with the average value again being calculated. Frequency of contact with friends was determined based on the average value for contact with close friends. Regarding social activities, respondents were asked to indicate the frequency of participation in seven types: attendance of alumni meetings, religious meetings, social clubs (in a senior center), sports clubs, lifelong education classes, voluntary activities, community welfare center activities. Frequency of all activities was measured on a 7-point scale ranging from 0 (never) to 6 (over four times a week), and the average score across all the activities was used in the analysis. Number of children, relatives, and friends were included in the total network size calculation.

#### 2.2.2. Depressive Symptoms

The Korean version of the Geriatric Depression Scale-Short Form (SGDS-K) was used to measure depressive symptoms. The SGDS was developed by Yesavage and Sheik [29] and translated into Korean by Bae and Cho [30]. The SGDS-K is composed of 15 items taken from the 30-item GDS-K. The SGDS-K has shown satisfactory reliability (Cronbach’s alpha = 0.90) and validity [31]. The Cronbach’s alpha in this study was 0.92.

#### 2.2.3. Covariates

Socioeconomic and health variables were the covariates in this study. The socioeconomic variables included age, education level, and equivalent household income, while SRH was the health variable. Education level was classified as elementary school or below, middle school, high school, and college or above. Equivalent household income was calculated as the total household income divided by the square root of the number of household members. These scores were then divided into tertiles. Self-rated health (SRH) was measured with a single question “How would you rate your health in general?” (1 = very poor, 5 = very good) and then coded dichotomously (0 = very good/good, 1 = fair/poor/very poor).

### 2.3. Data Analysis

Distributions and descriptive statistics were calculated for socioeconomic and health variables, as well as the variables used to distinguish network types. Two clustering techniques (hierarchical and *K*-means cluster analysis) were applied to the network types of men and women. Seven criterion variables were used in cluster analysis: marital status, living arrangements, frequency of contact with children, close friends, and close relatives, participation in social activities, and total network size. Before cluster analysis, the criterion variables were standardized to rule out any effect of the use of different scales [32]. Then, we carried out a hierarchical clustering procedure using Ward’s minimum variance method to determine the ideal number of clusters. Then, *K*-means cluster analysis was performed. In this analysis, initial cluster centers are assigned to each of the criterion variables, and the iteration of cluster centers is repeated until the prescribed number of optimal clusters is achieved based on distances between these cluster centers. After network clusters were derived, the distribution of criterion variables across clusters was assessed to determine the differences among the clusters and evaluate the validity of them [32]. The clusters were also compared in every background and health characteristics using chi-square test and analysis of variance (ANOVA). Finally, we conducted multiple regression analysis, stratified by gender, to estimate the effect of network type on depressive symptoms, while controlling for covariates. The diverse network type for men, the diverse-married type for women were used as the reference groups. The analysis used weighted data; the sampling weights were calculated based on a combination of design, non-response, and post-stratification weights [33].

## 3. Results

### 3.1. Participants’ Characteristics of Older Men and Women

There were differences between men and women on most of the variables (Table 1). The mean age of women (71.63 years) was older than that of men (72.93 years). The percentage of elementary school and less was higher among older women (50.1%) than older men (29.9%). More women reported poor self-rated health and lived alone than men (57.9% and 46.3%; 27.9% and 9.8% respectively). Women had more contacts with children, close friends/neighbors, and close relatives than men. More women participated in social activities, and provided instrumental support to adult children and caring support to spouse than men.

### 3.2. Intercorrelations of Profile Variables and Depressive Symptoms

Table 2 presents descriptive statistics and the intercorrelations of the profile variables and depressive symptoms. Marital status and living arrangements were relatively highly correlated (correlation coefficient *r* = −0.72), but other variables were uncorrelated.

### 3.3. Social Network Types of Older Men and Women

Table 3 shows the frequency and proportions of the criterion variables, respectively, for the five network types of older men and women.

The social network types of the older men were classified as diverse, social-activity-focused, or family focused, and there were also two restricted network subtypes: restricted-couple-focused and restricted-unmarried. The diverse network type was dominant in the total network size and the frequency of contacts with close friends. Individuals in this group engaged in a moderate amount of social activity and had relatively frequent contact with close relatives and children. The majority of individuals in this type of network were married (93.7%), and few lived alone (3.7%). Only 8.0% of the men (*n* = 190) were in a diverse network, which was thus the least prevalent of the five network types. In total, 22.7% of men (*n* = 542) were in a social-activity-focused network. Individuals in this group had the highest frequency of participation in social activities. The frequency of contact with close friends was moderate, while that with children and close relatives was low. The total network size of these individuals was smaller than that of the other men. These individuals were typically married (97.4%), and few lived alone (0.6%). In total, 32.0% of the men (*n* = 762) were in a family focused network, making it the dominant network type in Korean older men of this study. The majority of these individuals were married (96.7%) and none lived alone. The individuals in this type of network had the most contact with their children and close relatives, although their level of participation in social activities was low.

In total, 27.4% (*n* = 654) of the men were in a restricted-couple-focused network, making it the second most common network type, none of whom in this group lived alone. Individuals in a restricted-couple network were in moderately frequent contact with their children, but had the least contact with close friends and relatives, and the lowest level of participation in social activities and total network size. In total, 9.9% (*n* = 236) of the men were in a restricted-unmarried network; 93.2% of these men lived alone and 95.3% were unmarried. They had the least amount of contact with their children and, similar to those in a restricted-couple-focused network, also exhibited low scores for frequency of contact with close friends and relatives and frequency of participation in social activities. However, the individuals in the former type of network tended to have more social contact overall, except with their children than in a restricted-couple-focused type.

The social network types of the older women were classified into two diverse subtypes (diverse-married and diverse-unmarried), a family focused and two restricted subtypes (restricted-couple-focused, and restricted-unmarried).

In total, 13.9% (*n* = 310) of the women were in a diverse-married type network. The majority of the individuals in this type of network were married (96.1%) and a few lived alone (0.3%). They had the most contact with close friends and relatives, the highest rate of participation in social activities, and the largest total network size; the frequency of contact with children was also high. In total, 12.8% (*n* = 285) of the women were in a diverse-unmarried network, and 99.3% of women lived alone. The frequency of participation in social activities among the women in a diverse-unmarried network was as high as that of those in a diverse-married network. The frequency of contact with close friends and relatives was relatively high among individuals in the former type of network, but the frequency of contact with children was as low as that of individuals in a restricted-couple-focused network. The total network size (7.08 ± 3.09) of the women in a diverse-unmarried network was smaller only than that of those in a diverse-married network (9.47 ± 2.86), and thus was also higher than the average among females (5.75 ± 2.88).

In total, 19.2% (*n* = 427) of the women were in a family focused network; all of these women were married and lived with others, and their frequency of contact with children was highest among the network types. Most of these individuals lived with adult children (99.8%). However, aside from frequency of contact with children, these women tended to have less social contact overall than those in other network types.

The restricted-couple-focused network was dominant among the women (37.1%, *n* = 826). Women in this type of network had low scores for frequency of contact with children, close friends, and relatives, and for participation in social activities. The total network size was also below average. The restricted-unmarried network type was the least well-represented among the women (16.9%, *n* = 376). All individuals in this type of network were unmarried and most of them lived alone (98.1%); they also had the lowest scores for contact with children, close friends, and relatives, and participation in social activities.

### 3.4. Social Network Types according to Socioeconomic and Health-Related Variables

Social network types were also analyzed according to age, socioeconomic status, SRH, (Table 3).

A high proportion of men in diverse (22.1%) and social-activity-focused (23.6%) networks had higher education (college or above); the proportion of such men was lowest for the restricted-couple-focused network type (18.6%). About fifty percent of older men in the restricted-unmarried network were included in lowest equivalent household income. More men in the restricted-couple-focused and the restricted-unmarried networks had poor SRH (51.2% and 52.1%, respectively) than in the other network types.

The proportion of women with higher education (college or above) was highest in the diverse-married type network (9.1%) and lowest in the restricted-unmarried type network (4.0%). The proportion of poor SHR (68.9%) was largest among the women in the restricted-unmarried network, and lowest among those in the diverse-married network (48.1%). Regarding equivalent household income, the proportion of women in the lowest income category was very large in the restricted-unmarried network (62.8%) and lowest in family focused network (11.5%).

### 3.5. The Relationship of Social Network Types to Depressive Symptoms

Table 4 presents the scores of depressive symptoms according to social network type and gender, and results of the multiple regression analysis, which was controlled for covariates. The score of depressive symptoms was 2.62 ± 3.24 in men and 3.10 ± 3.58 in women. The men in the restricted-unmarried network (3.90 ± 3.97), and women in the restricted-unmarried network (4.54 ± 4.26), were more likely to exhibit depressive symptoms than those in any other network. The men in the diverse (1.95 ± 2.86) or family focused (2.22 ± 2.92) network were the least likely to show depressive symptoms. Among the women, the score of depressive symptoms was lowest in the diverse-married (1.85 ± 2.57) and diverse-unmarried (2.36 ± 3.07) networks.

All of the restricted type networks were significantly associated with a higher risk of depressive symptoms in both men and women. Men and women in the restricted-unmarried networks, respectively, were more likely to experience depressive symptoms (*ß* = 0.14, *ß* = 0.19 respectively). Restricted-couple-focused networks was significantly associated with depressive symptoms in men (*ß* = 0.14) and women (*ß* = 0.11). Family focused network was associated with depressive symptoms in women (*ß* = 0.14) but not in men.

## 4. Discussion

Our study identified five different types of social network in men and women. In addition, those in restricted-couple-focused and restricted-unmarried network types were more likely to report depressive symptoms in both men and women. Older women in family focused network experienced higher level of depressive symptoms, but older men did not.

Among identified network types, the restricted network types (restricted couple focused and restricted unmarried) were also most prevalent for both older men and women in Korea, which is opposite to those previously reported in other Asian countries (e.g., China and Japan) [12,13]. This difference may in part be attributed to the radical changes in Korean society associated with industrialization. Until recently, South Korea shared a Confucian culture with Japan and China. These countries have undergone dynamic socioeconomic transformations in association with economic growth and urbanization. However, unlike China and Japan, placing a high value on family life and residence with children are still prevalent among older adults, albeit to a lesser extent than before due to westernization [12,34,35], the radical transition to an industrialized, modern society in Korea resulted in a rapid shift from traditional social norms based on family obligations to the Western norms of individualism and independence [36,37]. Compared to other Asian countries, coresidence with older parents, and the view that their care is the child’s responsibility, have significantly declined in South Korea, and the percentage of older adults living with their children decreased from 54.7% in 1994 to 23.7% in 2017 [33]. Moreover, 77.4% of older Korean adults reported that they did not want to live with their adult children [24]. In recent Korean studies [14,15], the family focused network type was shown to no longer be prevalent.

Specifically, we found differences in dominant network type between older men and women. The couple-focused network was dominant for older women (37.1%), while in older men, family focused type was (32.0%). Despite women’s superior ability to maintain more relationships and diverse social connections, in this study more women (54.0%) belonged to a restricted network (restricted-couple-focused or restricted-unmarried) than men (restricted-couple-focused or restricted-unmarried; 37.3%). This is in line with previous Korean studies, in which men were more likely to have diverse or family centered networks [14,15,38], and Korean older women had the highest proportions of a restricted type [38]. In this study, the proportion of women in a restricted-unmarried network (16.9%) was larger than that of men (9.9%). The increased number of elderly women living alone, likely due to their longer life expectancy, in part explains this difference in the number of older adults with restricted social relations as widowers or widows.

The social-activity-focused network identified in this study for some of the older men is novel, and is characterized by a high level of participation in various social activities, such as education programs for seniors, volunteer work, and religious meetings. Older men in a social-activity-focused network tended to have a higher level of education (college or above) and higher economic status than those in restricted networks, including the family focused subtype. Older adults with active social lives are more likely to be male [39], and are also younger [39,40] and better educated [39,40,41]. Moreover, they have a higher economic status [20], which together with the change in household type from extended to nuclear family may provide them with more opportunities to participate in social activities outside the family [14].

Consistent with several previous studies reporting an association between limited social ties and poorer health [7,14,42], the older men and women in restricted networks in this study had more depressive symptoms. The men in restricted-couple-focused and restricted-unmarried social networks tended to have lower educational and economic status, and also exhibited poorer SRH and less participation in economic activities than those in other types of network, which previous studies have identified to be associated with depressive symptoms in older men and women [43,44]. Interestingly, compared to older men in the restricted-couple-focused network, those in the restricted-unmarried network had more depressive symptoms, even though they participated in more social activities and had more contact with friends and relatives. Most of the older men in the restricted-unmarried network had no spouse and lived alone; these individuals also had the least contact with children. Men primarily rely on their spouses for intimacy and social contact, as well as for emotional and caregiver support [1,45]. Thus, in the event of the death of their spouses, men lose their major source of emotional and social support [46,47]. The presence of a spouse may therefore be particularly important for preventing depressive symptoms in older men.

Another interesting finding was that older women in the diverse-unmarried type network reported better mental health than those in restricted-couple-focused and family focused networks, even though they lived alone and had lower educational and income levels, as well as poorer SRH. Moreover, compared to men in the family focused network, women in the family focused network were more likely to exhibit depressive symptoms. This accords with a previous study [24] reporting that living alone appears to be beneficial to older Korean women, and does not promote depressive symptoms. In a longitudinal health study of women aged 60 to 72 years, those living alone did not have poorer mental health, and were not more socially isolated [17]. In this study, women in the restricted-couple-focused network were the most likely to have a spouse with poor SRH, and to be caring for their spouse. In total, 99.8% of the women in the family focused network coresided with their adult children, and most of them provided instrumental support to their children (91.1%). Moreover, 11.2% of them were rearing grandchildren, which was the highest rate among all network types. Meanwhile, women in diverse-married and diverse-unmarried networks were less likely to have a spouse with poor SRH, or to coreside with children, than those in any of the restricted network types and family focused type. In the traditional Confucian Korean society, a wife lived with her husband in the house of his parents, and spent much of her time caring for the entire household. In turn, her coresident son and daughter-in-law cared for her in old age. However, with the modernization and industrialization of Korean society, women have become more socially active, and also participate more in the labor force [48]. However, public sector support with respect to childcare for working women has been lacking, with the extended family system thus still serving as the major source of support [49]. Therefore, older women tend to shoulder the burden of caring not only for their grandchildren and spouse, but must also provide instrumental support as the daughter-in-law is typically at work. While the cultural and social changes that have taken place in Korea have provided younger women with more opportunities for education and employment, older women remain burdened by the expectation that they will provide support.

This study had some limitations. First, we used a cross-sectional design, so the relationship of social network type with mental health should be interpreted with caution. Longitudinal data should be analyzed to clarify this association in older adults, stratified by gender. Second, all of the criterion variables were specifically concerned with structural aspects of social relationships, although social network function and quality also contribute significantly to well-being in old age. Further studies are therefore needed. Last, we identified different network types in older men and women in this study, but did not examine the gender differences among social network types. Further research would be needed to test whether network types are different from one another across genders. Despite these limitations, our study also had some important strengths. To our knowledge, it is the first study to identify social network topologies by gender among older adults in South Korea. Network typology can be a useful and practical instrument for identifying older adults at risk in community. The findings contribute to understanding of social network types of older men and women in later life and their implications for health and wellbeing. The results suggest that there are differences in those with poor networks by gender, and interventions for them should be different by gender. Moreover, we analyzed weighted data from a nationally representative sample, ensuring the generalizability of the findings. In addition, we controlled for various factors that may influence the maintenance of social relations, such as functional limitations and cognitive impairment.

## 5. Conclusions

This study provides evidence that social network types, and their impact on mental health, differ between men and women. Additional empirical studies conducted in other cultural settings should be performed to validate our findings.

## Figures and Tables

**Table 1:** Characteristics of the social network criterion, socioeconomic and health -related variables of older men (*n* = 2384) and women (*n* = 2224) in the 2017 Korean Living Profile Survey of Older People.

	All	Men	Women	
	*n* (%) or Mean ± SD	*n* (%) or Mean ± SD	*n* (%) or Mean ± SD	
*n*	4608	2384	2224	
Criterion variables of social network type				
Married	3406	2083 (86.8)	1323 (60.4)	**
Living alone	884	231 (9.8)	653 (27.9)	**
Frequency of contact with children	5.24 ± 2.93	5.15 ± 2.87	5.34 ± 2.98	*
Frequency of contact with close friends/neighbors	4.47 ± 1.34	2.34 ± 0.79	2.41 ± 0.71	**
Frequency of contact with close relatives	1.40 ± 0.77	1.31 ± 0.73	1.49 ± 0.80	**
Frequency of participation in social acitivies	4.30 ± 3.38	2.55 ± 2.88	3.72 ± 3.21	**
Total network size	5.71 ± 3.03	5.66 ± 3.17	5.76 ± 2.88	
Depressive symptoms	2.86 ± 3.41	2.63 ± 3.24	3.10 ± 3.58	**
Covariates				
Age	72.30 ± 5.33	72.93 ± 5.50	71.63 ± 5.05	** **
65–74	3101 (67.3)	1492 (68.2)	1609 (77.4)	
75–84	1416(30.7)	827 (28.6)	588 (21.1)	
85 and over	92(2.0)	65 (3.2)	27 (1.5)	
Education				
Elementary school and less	1934 (42.0)	753 (29.9)	1180 (50.1)	**
Middle school	1026 (22.3)	553 (23.4)	473 (22.0)	
High school	1142 (24.8)	702 (30.3)	440 (21.1)	
College and over	507 (11.0)	376 (16.4)	131 (6.9)	
Equivalent household income ^a^				
1st 33.3%	1278 (27.7)	701 (32.2)	576 (28.6)	**
2nd 33.3%	1754 (38.1)	926 (38.4)	828 (37.6)	
3rd 33.3% (lowest)	1577 (34.2)	757 (29.5)	820 (33.8)	
Self-rated health				
Good	2194 (47.6)	1274 (53.7)	920 (42.1)	**
Bad	2414 (52.4)	1110 (46.3)	1304 (57.9)	

* *p* < 0.05; ** *p* < 0.01 for difference between men and women; SD = standard deviation. ^a^ Monthly household income divided by the square root of the number of household members.

**Table 2:** Intercorrelation of social network profile variables and depressive symptoms.

	1	2	3	4	5	6	7	8
1. Married	1							
2. Living alone (%)	−0.72 **	1						
3. Frequency of contact with children	0.009	0.33 **	1					
4. Frequency of contact with close friends/neighbours	−0.037	−0.017	−0.049 *	1				
5. Frequency of contact with close relatives	−0.041	0.032	0.033	0.162 **	1			
6. Frequency of participation in social activities	0.013	−0.074 **	−0.097 **	0.309 **	0.103 **	1		
7. Total network size	−0.053 *	0.041	0.085 **	0.204 **	0.191 **	0.193 **	1	
8. Depressive symptoms	0.107 **	−0.089 **	−0.041	−0.220 **	−0.162 **	−0.184 **	−0.230 **	1

* *p* < 0.05; ** *p* < 0.01.

**Table 3:** Characteristics of the social network criterion, socioeconomic and health-related variables for each of the five social network types of older men (*n* = 2384) and women (*n* = 2224) in the 2017 Korean Living Profile Survey of Older People.

	Men						Women					
	Diverse ^a^	Social Activity -Focused ^b^	Family-Focused ^c^	Restricted-Couple-Focused ^d^	Restricted-Unmarried ^e^	*p*	Diverse-Married ^a^	Diverse-Unmarried ^b^	Family-Focused ^c^	Restricted-Couple-Focused ^d^	Restricted-Unmarried ^e^	*p*
	*n* (%) or Mean ± SD	*n* (%) or Mean ± SD	*n* (%) or Mean ± SD	*n* (%) or Mean ± SD	*n* (%) or Mean ± SD		*n* (%) or Mean ± SD	*n* (%) or Mean ± SD	*n* (%) or Mean ± SD	*n* (%) or Mean ± SD	*n* (%) or Mean ± SD	
N =	190 (8.0)	542 (22.7)	762 (32.0)	654 (27.4)	236 (9.9)		310 (13.9)	285 (12.8)	427 (19.2)	826 (37.1)	376 (16.9)	
Criterion variables of social network type												
Married	178 (93.7)	528 (97.4)	737 (96.7)	629 (96.2)	11 (4.7)	*	298 (96.1)	0	215 (50.4)	820 (98.1)	0	*
Living alone	7 (3.7)	3 (0.6)	0	0	220 (93.2)	*	1 (0.3)	283 (99.3)	0 (0)	0 (0)	369 (98.1)	*
Frequency of contact with children	5.62 ± 2.98	4.84 ± 2.44	5.70 ± 2.91	5.38 ± 3.03	3.00 ± 1.75	a,c > b,d > e **	4.47 ± 1.43	4.04 ± 1.36	10.71 ± 1.32	4.07 ± 1.23	3.46 ± 1.79	c > a > b,d > e **
Frequency of contact with close friends/neighbours	2.81 ± 0.50	2.73 ± 0.57	2.77 ± 0.51	1.71 ± 0.71	2.14 ± 0.82	a, b,c > e>d **	2.89 ± 0.47	2.75 ± 0.47	2.26 ± 0.74	2.26 ± 0.65	1.83 ± 0.70	a > b > c,d > e **
Frequency of contact with close relatives	1.54 ± 0.63	1.24 ± 0.65	1.80 ± 0.70	0.94 ± 0.52	1.17 ± 0.76	a, c > b,e > d **	1.90 ± 0.77	1.79 ± 0.77	1.47 ± 0.81	1.32 ± 0.69	0.98 ± 0.70	a > b > c,d > e **
Frequency of participation in social acitivies	3.43 ± 3.30	6.33 ± 2.52	1.43 ± 1.55	1.15 ± 1.53	2.22 ± 2.76	b > a > c,e > d **	5.64 ± 3.54	5.63 ± 3.50	2.85 ± 2.77	2.61 ± 2.35	2.43 ± 2.19	b > a > c,d > e **
Total network size	13.76 ± 3.33	5.29 ± 2.23	6.12 ± 2.19	4.24 ± 1.80	4.91 ± 2.96	a > b, c > d,e **	8.41 ± 3.04	6.65 ± 2.99	5.50 ± 2.30	4.59 ± 1.89	3.97 ± 2.04	a > b > c > d,e **
Covariates												
Age	72.98 ± 5.42	73.60 ± 5.32	71.97 ± 5.09	73.43 ± 5.85	73.10 ± 5.84	*	70.81 ± 4.38	73.28 ± 5.04	71.08 ± 5.43	70.83 ± 4.70	73.41 ± 5.55	*
Education												
Elementary school and less	49 (25.8)	136 (25.1)	259 (34.0)	227 (34.7)	82 (34.8)	*	129 (41.6)	173 (60.7)	240 (56.2)	413 (50.0)	225 (59.8)	*
Middle school	45 (23.7)	119 (22.0)	206 (27.0)	134 (20.5)	49 (20.8)		63 (20.3)	50 (17.5)	83 (19.4)	198 (24.0)	79 (21.0)	
High school	54 (28.4)	159 (29.3)	212 (27.8)	216 (33.0)	61 (25.8)		90 (29.0)	44 (15.4)	80 (18.7)	169 (20.5)	57 (15.2)	
College and over	42 (22.1)	128 (23.6)	85 (11.2)	77 (11.8)	44 (18.6)		28 (9.1)	18 (6.3)	24 (5.6)	46 (5.6)	15 (4.0)	
Equivalent household income ^f^												
1st 33.3%	68 (35.8)	186 (34.3)	250 (26.9)	157 (24.0)	40 (16.9)	*	100 (32.3)	40 (14.0)	226 (52.9)	181 (21.9)	29 (7.7)	*
2nd 33.3%	69 (36.3)	207 (38.2)	307 (40.3)	263 (40.2)	80 (33.9)		119 (30.4)	115 (40.4)	152 (35.6)	331 (40.1)	111 (29.5)	
3rd 33.3% (lowest)	53 (27.9)	149 (27.5)	205 (32.8)	234 (35.8)	116 (49.2)		91 (29.4)	130 (45.6)	49 (11.5)	314 (38.0)	236 (62.8)	
Self-rated health												
Good	108 (56.8)	314 (57.9)	420 (55.1)	319 (48.8)	113 (47.9)	*	161 (51.9)	123 (43.2)	173 (40.5)	346 (41.9)	117 (31.1)	*
Bad	82 (43.2)	228 (42.1)	342 (44.9)	335 (51.2)	123 (52.1)		149 (48.1)	162 (56.8)	254 (59.5)	480 (58.1)	259 (68.9)	

* *p* < 0.01 for difference for levels of each variable; ** *p* < 0.01 for Scheffe test for differences among the five social network types.; SD = standard deviation. ^f^ Monthly household income divided by the square root of the number of household members.

**Table 4:** Scores of depressive symptoms and multiple regression for depressive symptoms among older men (*n* = 2384) and women (*n* = 2224) in the 2017 Korean Living Profile Survey of Older People.

Variable	N	Depressive Symptoms		B (SE) ^a^	*ß* ^a^
		Mean ± SD	*p*		
Men	2384	2.62 ± 3.24			
Social Network Types of men					
Diverse	190	1.95 ± 2.86	*p* < 0.01	ref	
Social activity-focused	542	1.94 ± 2.76		−0.12 (0.24)	−0.12
Family-focused	762	2.22 ± 2.92		0.11 (0.23)	0.02
Restricted-couple-focused	654	3.40 ± 3.48		1.04 (0.24)	0.14 *
Restricted-unmarried	236	3.90 ± 3.97		1.53 (0.28)	0.14 *
Women	2224	3.10 ± 3.58			
Social Network Types of women					
Diverse-married	310	1.85 ± 2.57	*p* < 0.01	ref	
Diverse-unmarried	285	2.36 ± 3.07		0.06 (0.27)	0.01
Family-focused	427	3.29 ± 3.65		1.25 (0.24)	0.14 *
Restricted-couple-focused	826	3.06 ± 3.44		0.81 (0.22)	0.11 *
Restricted-unmarried	376	4.54 ± 4.26		1.79 (0.25)	0.19 *

* *p* < 0.01; B = unstandardized coefficient; SE = standard error; *ß* = standardized coefficient.; SD = standard deviation.; ref = reference group. ^a^ adjusted by age, educational level, equivalent household income, and self-rated health.
